# Three-dimensional visualization and evaluation of hilar cholangiocarcinoma resectability and proposal of a new classification

**DOI:** 10.1186/s12957-023-03126-2

**Published:** 2023-08-05

**Authors:** Jun-Zhe Zhang, Chuan-Xin Yang, Si Gao, Jun-Feng Bu, Qin-Qin Li, Hao-Lu Wang, Kai-Ni Yang, Shan-Shi Tong, Li-Jun Qian, Jin Zhang, Rong Hua, Yong-Wei Sun, Jia-Yan Yan, Wei Chen

**Affiliations:** 1 Department of Biliary-Pancreatic Surgery, Renji Hospital, School of Medicine, Shanghai Jiaotong University, Shanghai, 200127 People’s Republic of China; 2 Department of Hepatobiliary and Pancreatic Surgery, Shanghai Sixth People’s Hospital Affiliated to Shanghai Jiao Tong University School of Medicine, Shanghai, 200233 People’s Republic of China; 3 Department of Pathology, Ruijin Hospital, Shanghai Jiao Tong University School of Medicine, Shanghai, 200025 People’s Republic of China; 4 University of Queensland Diamantina Institute, University of Queensland, Woolloongabba, QLD 4102 Australia; 5 Department of Radiology, Renji Hospital, School of Medicine, Shanghai Jiaotong University, Shanghai, 200127 People’s Republic of China; 6 Department of Liver Surgery, Liver Cancer Institute, Zhongshan Hospital, Fudan University, Key Laboratory of Carcinogenesis and Cancer Invasion, Ministry of Education, Shanghai, 200032 People’s Republic of China

**Keywords:** Hilar cholangiocarcinoma, Biliary surgical procedure, Three-dimensional image, Classification

## Abstract

**Background:**

As digital medicine has exerted profound influences upon diagnosis and treatment of hepatobiliary diseases, our study aims to investigate the accuracy of three-dimensional visualization and evaluation (3DVE) system in assessing the resectability of hilar cholangiocarcinoma (hCCA), and explores its potential clinical value.

**Materials and methods:**

The discovery cohort, containing 111 patients from April 2013 to December 2019, was retrospectively included to determine resectability according to revised criteria for unresectability of hCCA. 3D visualization models were reconstructed to evaluate resectability parameters including biliary infiltration, vascular involvement, hepatic atrophy and metastasis. Evaluation accuracy were compared between contrast-enhanced CT and 3DVE. Logistic analysis was performed to identify independent risk factors of R0 resection. A new comprehensive 3DVE classification of hCCA based on factors influencing resectability was proposed to investigate its role in predicting R0 resection and prognosis. The main outcomes were also analyzed in cohort validation, including 34 patients from January 2020 to August 2022.

**Results:**

3DVE showed an accuracy rate of 91% (95%CI 83.6–95.4%) in preoperatively evaluating hCCA resectability, significantly higher than 81% (95%CI 72.8–87.7%) of that of CT (*p* = 0.03). By multivariable analysis, hepatic artery involvement in 3DVE was identified an independent risk factor for R1 or R2 resection (OR = 3.5, 95%CI 1.4,8.8, *P* < 0.01). New 3DVE hCCA classification was valuable in predicting patients’ R0 resection rate (*p* < 0.001) and prognosis (*p* < 0.0001). The main outcomes were internally validated.

**Conclusions:**

3DVE exhibited a better efficacy in evaluating hCCA resectability, compared with contrast-enhanced CT. Preoperative 3DVE demonstrated hepatic artery involvement was an independent risk factor for the absence of R0 margin. 3DVE classification of hCCA was valuable in clinical practice.

**Supplementary Information:**

The online version contains supplementary material available at 10.1186/s12957-023-03126-2.

## Background

Hilar cholangiocarcinoma (hCCA), arising from the common hepatic duct or first-class hepatic ducts, is a biliary malignancy with an incidence less than 1 per 100,000 men in the most countries [1, 2]. As previously reported, a 5-year survival rate ranging from 18.9 to 38.1% attests to the refractory nature of hCCA [3–6]. Radical resection remains the only hope for potential cure of hCCA, but its success rate has been limited to 42 to 70.9% due to several unfavorable factors, including caudate lobe involvement, longitudinal extension along hepatic ducts (biliary infiltration), radial invasion into periductal tissues, distant metastasis, and insufficient future liver remnant (FLR) [1, 6–8]. Thus, a precise preoperative evaluation of resectability is vital to facilitate appropriate therapeutic strategies and prevent unnecessary laparotomy for hCCA patients.

To assess biliary infiltration, endoscopic retrograde cholangiography (ERC), percutaneous transhepatic cholangiography (PTC), and magnetic resonance cholangiopancreatography (MRCP) yielded accuracy rates of 87%, 40–90%, and 80–95%, respectively, which are superior to 80% of contrast-enhanced computed tomography (CT) [9–12]. Whereas, ERC, PTC, and MRCP mainly considered biliary infiltration without other resectability factors. For the evaluation of hepatic artery (HA) and portal vein (PV) involvement, contrast-enhanced CT, and magnetic resonance imaging (MRI) exhibited accuracy rates of 77–93% and 73–80%, respectively [10, 13, 14].

Three-dimensional visualization and evaluation (3DVE) system is a digital image processing technology used for hepatobiliary diseases [15–17]. 3DVE integrates the raw images of CT or MRI, reconstructs the lesion, vessels and complex liver anatomy, then finally formulates a stereoscopic model that comprehensively displays the spatial relationship between lesions and adjacent tissues [17–19]. Prior studies have demonstrated the great value of 3DVE in evaluating biliary strictures, as well as HA variations, based on the new classification and nomenclature system of HA, named the CRL system [1, 15, 19]. Additionally, 3D imaging and the derived tools, such as 3D printing and virtual hepatectomy, helped to intuitively visualize the portal territories of liver and calculate FLR volume, which facilitate radical hepatectomy, even in patients with impaired liver function or tumor located in challenging positions [20, 21]. To date, there have been few studies on the use of 3D visualization technology in evaluating resectability of hepatobiliary malignancy, including hCCA [5, 9, 10, 13, 20]. Furthermore, a practical and feasible 3DVE classification can potentially add value to clinical decision-making for hCCA.

Here, we explore the efficacy of 3DVE in assessing hCCA resectability by comparing with that of CT. In addition, we proposed a new 3DVE classification of hCCA and explored its clinical value in predicting surgical outcomes and prognosis.

## Materials and methods

**Fig. 1:**
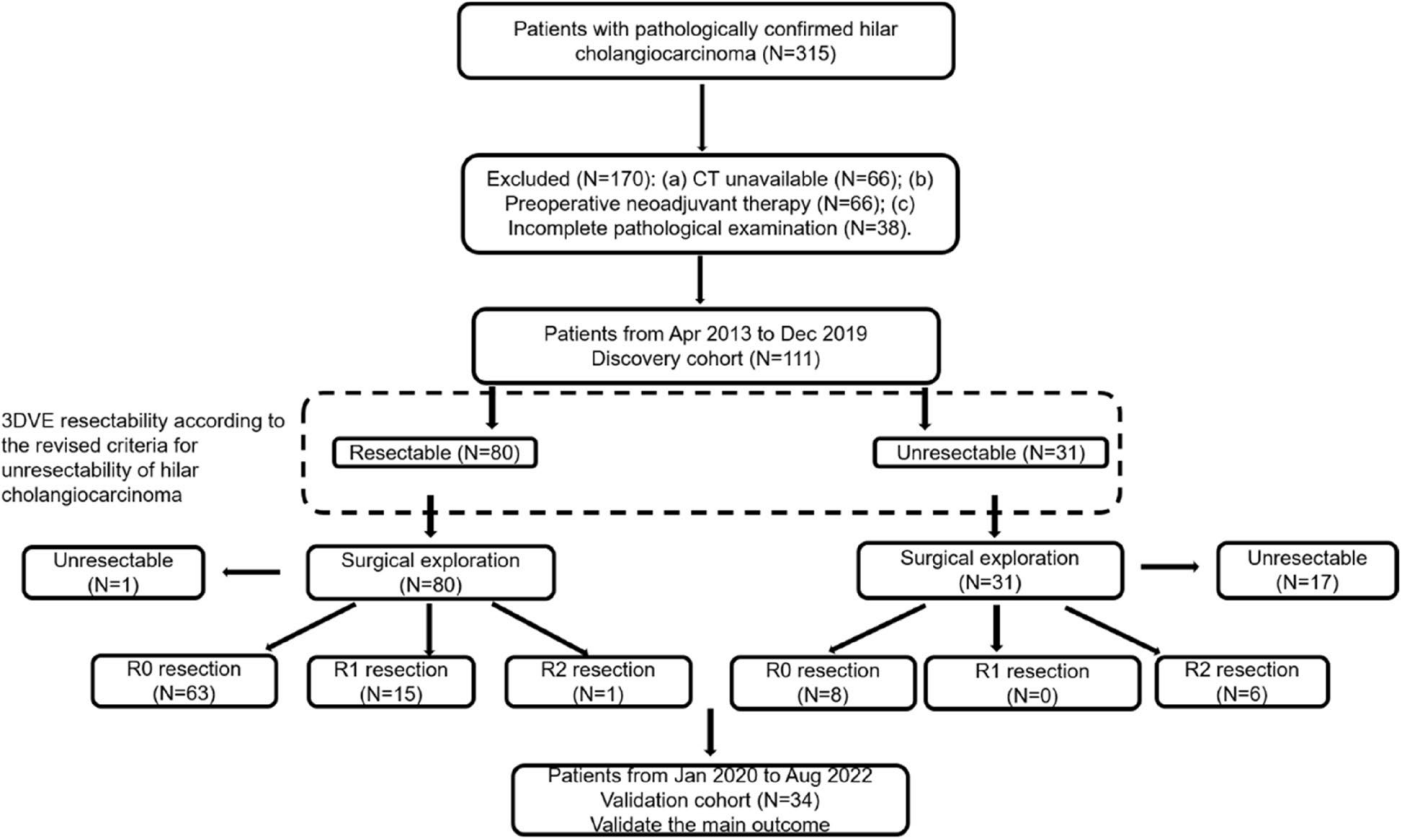
Flowchart show the study population. 3DVE resectability was evaluated according to revised criteria for unresectability of hilar cholangiocarcinoma

The study flowchart was shown in Fig. 1.

### Patient selection

The institutional electronic patient database was searched for patients pathologically confirmed hCCA, with the following inclusion criteria: (a) underwent preoperative contrast-enhanced CT and 3DVE; (b) underwent full surgical exploration.

### Imaging technique

All the patients held their breath in full inspiration before scanning and were instructed to do so during each scan phase. A preoperative triple-phase CT was taken using a 64-slice CT scanner (LightSpeed VCT 64, GE Healthcare, Milwaukee) following the standard protocol that involved a slice thickness for axial images of 1.25 mm, a reconstruction slice thickness of 1.25 mm, and a reconstruction interval of 1.25 mm. After administration of nonionic iodinated contrast material (Iopamidol 370 mg/mL; Bracco Sine Pharmaceutical Co., Ltd.) with an injection rate of 3–4 mL/s, the arterial and portal venous phase scans were acquired in 20–25 s and 60–65 s, respectively. The contrast-enhanced CT data were sent to the picture archiving and communication system (PACS) for interpretation.

CT data in DICOM format were transferred to an IQQA-LIVER workstation (EDDA Technology Inc., Princeton). The 3D visualization processing was structured as a five-step procedure. Firstly, imported contrast-enhanced CT data were automatically processed into an original 3D liver model. Secondly, the liver contour was constructed in portal venous phase by the semi-automated seeded region growing segmentation technique and the active contour was manually modified. Thirdly, the HA was automatically extracted based on the contrast agent in the arterial phase and PV and hepatic veins were extracted from the portal venous phase. Dilated bile ducts, seen as hypodensity structures in the portal venous phase, were extracted by reverse processing, and 3D bile duct images were rendered. The course, morphology and continuity of blood vessels and biliary tract were checked, and manual revision was necessary if branches were wrongly reconstructed. Then, the vasculature structures from two phases were registered and merged. Fourthly, a seed point was selected in the center of the tumor and the borders of the tumor were manually outlined in coronal, sagittal, and horizontal planes, respectively (Figure E1). Finally, FLR was estimated and a virtual liver resection was performed (Figure E2).

### Image analysis

All contrast-enhanced CT images were retrospectively reviewed in consensus with a hepatopancreatobiliary surgeon (W.C) and an abdominal radiologist (L.J.Q). The radiologists reviewed the CT images on the PACS. The three-dimensional visualization evaluation (3DVE) was collaboratively accomplished by two hepatopancreatobiliary surgeons (J.Y.Y, C.X.Y) and an abdominal radiologist (J.Z). The 3D visualization images were viewed and assessed on the IQQA-LIVER workstation. All observers were aware of the diagnosis of hCCA but were blinded to other clinicopathological features of the patients, the agreement value (κ value) of observers were calculated. After the first image analysis, an experienced doctor (Rong, Hua) reviewed the discordant findings between observers to decide which result to use.

The following imaging features were qualitatively analyzed for resectability evaluation. Biliary infiltration was considered present if (a) the ductal wall was hyperattenuating compared with the liver in the portal venous phase, (b) if there was irregular ductal wall thickening with an asymmetric upstream intrahepatic ductal dilation, or (c) if the lumen was obliterated by intraductal soft-tissue [22, 23]. The status of biliary infiltration referred to the Bismuth-Corlette classification (Figure E3) [24]. The criteria for vascular involvement of tumor included vessel occlusion, stenosis, contour deformity, and tumor encasement of the vessels (> 180° of circumferential involvement) [22, 25]. Hepatic lobar atrophy was defined as a reduction in the size of that lobe by at least 50%, or the lobe with portal hypoperfusion and dilatated hepatic ducts [26, 27]. Lymph node metastasis was considered positive if the lymph nodes were enlarged with central necrosis, larger than 10 mm in shortest diameter, or if its attenuation or signal density was greater than that of liver parenchyma in the portal venous phase [13, 22].

### Revised criteria for the unresectability of hilar cholangiocarcinoma

We revised the criteria for the unresectability of hCCA by combining the criteria proposed by Jarnagin [26] and Lee [13]. The revised criteria for unresectability included bilateral tumor extension to the limits of hepatic ductal dissection, which included the P point (located at the bifurcation of the right anterior and posterior branches of PV. This refers to the limit of right hepatic ductal dissection when left hemihepatectomy performed) and the U point (located at the umbilical portion of the left PV. This refers to the limit of left hepatic ductal dissection when right hemihepatectomy performed) (Figure E4) [28]. Tumor invasion of the main portal vein or proper hepatic artery, invasion or atrophy of one hepatic lobe with contralateral vascular invasion or contralateral tumor extension to the limit of hepatic ductal dissection, and tumor extension to the unilateral limit of hepatic ductal dissection with contralateral vascular invasion. Additional criteria for unresectability included insufficient FLR in virtual hepatectomy, metastasis to periaortic, pericaval, superior mesenteric artery, celiac lymph nodes, or the presence of distant metastasis.

### Surgical, pathological, clinical, and follow-up data collection

Liver resection procedures mainly involved minor hepatectomy, hemihepatectomy, trisegmentectomy, and specific hepatectomy. Lymphadenectomy was performed for clearance of all the lymph nodes of the 8th, 12th, and 13th groups. The operative approach of major hepatectomy referred to previous studies [26, 29]. Taj Mahal liver parenchymal resection and high hilar resection were the selective procedures for minor hepatectomy [30, 31]. Following exposure of the biliary confluence and evaluation for vascular involvement intraoperatively, pathological examination was performed on serial sections of the resection margin. Surgical and pathological resection margin status was categorized as negative (R0), microscopically positive (R1), or as the presence of gross residual tumor at surgery (R2). The full exploration was followed by curative or palliative surgical procedures to assess lymph and distant metastasis. The patients’ medical records, operative reports, and pathological reports were reviewed. The primary outcome, the R0 resection and survival information, was obtained by telephone.

### Statistical analysis

Categorical variables were compared using the *χ*^2^ test, and continuous variables were compared using an unpaired two-sided *t* test. The associations between ordinal categorical variables and dichotomous variables were analyzed using the Cochran-Armitage trend test. Cohen’s Kappa analysis was calculated, and κ values < 0 indicated no agreement and 0 < κ ≤ 0.2 slight, 0.2 < κ ≤ 0.4 fair, 0.4 < κ ≤ 0.6 moderate, 0.6 < κ ≤ 0.8 substantial, and 0.8 < κ ≤ 1 were almost perfect agreement. Ordinal categorical variables were compared using the Spearman rank correlation analysis. Multivariable logistic regression analysis was performed using the backward likelihood ratio method, including all variables with *p* < 0.1 in univariate analysis. *P* < 0.05 was considered statistically significant. A Kaplan–Meier curve was generated and Logrank test was performed for survival analysis. The statistical analysis was conducted using the SPSS version 19.0 software (IBM, Armonk) and GraphPad Prism version 8.0.2.

## Results

Of 315 eligible patients from April 2013 to August 2022, 170 were excluded due to one of the following reasons: (a) an interval greater than 6 weeks between CT imaging and surgery, and poor CT quality (*n* = 66); (b) underwent neoadjuvant therapy before surgery (*n* = 66); (c) an incomplete pathological examination that influenced the adjudgment of resectability (*n* = 38). The discovery cohort contained 111 patients from April 2013 to December 2019, the other 34 patients from January 2020 to August 2022 formed validation cohort.

### Characteristics of the discovery cohort

The discovery cohort consisted of 111 patients (68 men, 43 women) with a mean age of 65 years ± 10 (range, 31–89 years). The clinical classification is shown in Table E1, and the surgical procedures are in Table E2. In 3DVE resectable group, curative-intent surgery was performed in 79 patients and 1 patient was unresectable due to distant metastasis. The other 31 patients were diagnosed as unresectable cases by 3DVE due to invasion of the main PV or proper HA (*n* = 10), bilateral tumor extension to the limits of hepatic duct (*n* = 1), one hepatic lobe with contralateral vascular invasion (*n* = 1), tumor extension to unilateral dissection limit of bile duct and contralateral vascular invasion (*n* = 5), metastasis to periaortic, pericaval, superior mesenteric artery, or celiac lymph nodes (*n* = 9), distant metastasis (*n* = 4) and insufficient FLR volume (*n* = 1). According to the surgical findings, curative-intent surgery was attempted in 14 patients, and 17 patients were unresectable due to invasion of the main PV or proper HA (*n* = 12), bilateral tumor extension to the limits of hepatic duct (*n* = 2), distant metastasis (*n* = 1), and insufficient FLR volume (*n* = 2). According to revised unresectability criteria for hCCA, we found that HA involvement, PV involvement, hepatic lobar atrophy, lymph node metastasis and distant metastasis were more frequently observed in 3DVE unresectable group (*P* < 0.05) (Table 1).

**Table 1:** Clinicopathological features of 111 patients of hilar cholangiocarcinoma

	Resectability by three-dimensional visualization evaluation			
Clinicopathological features	Total	Resectable group	Unresectable group	*P* value
No. of patients	111	80	31	
Age (years)^a^	65 ± 10	66 ± 9	62 ± 11	0.053
Sex (M/F)	68/43	51/29	17/14	0.387
Preoperative biliary drainage	20/91	17/63	3/28	0.155
Tumor size(≤ 1/1 ~ 3 cm/ ≥ 3 cm	8/45/58	6/35/39	2/10/19	0.488
Tumor form				0.016
Sclerosing	43	35	8	
Mass	50	32	18	
Polypoid	9	9	0	
Mixed	9	4	5	
Tumor differentiation (well/moderately/poorly)	3/80/28	2/59/19	1/21/9	0.818
Hepatic artery involvement^b^	57(52.3%)	34(42.5%)	23(79.3%)	< 0.001
Portal vein involvement^c^	55(50.0%)	31(38.6%)	24(80.0%)	< 0.001
Hepatic vein involvement	5(4.5%)	3(3.8%)	2(6.5%)	0.538
Lymph node metastasis^d^	54(49.1%)	31(38.8%)	23(76.7%)	< 0.001
Distant metastasis	5(4.5%)	1(1.3%)	4(12.9%)	0.008
Perineural invasion	56(50.5%)	35(43.8%)	21(67.7%)	0.023
Hepatic lobe atrophy	21(18.9%)	10(12.5%)	11(35.5%)	0.006
Resection status				< 0.001
R0	71(64.9%)	63(78.8%)	8(25.8%)	
R1	15(13.5%)	15(18.8%)	0(0.0%)	
R2	7(6.3%)	1(1.2%)	6(19.4%)	
Unresectable	18(16.2%)	1(1.2%)	17(54.8%)	

Data are presented as number (%) or mean ± standard deviation

^a^Data are mean ± standard deviation

^b^2 cases belonged to unresectable group but lost the information of hepatic arterial involvement

^c^1 case belonged to unresectable group but lost the information of portal vein involvement

^d^1 case belonged to unresectable group but lost the information of lymph node metastasis

### 3DVE showed a better performance in assessing hilar cholangiocarcinoma resectability than CT

**Fig. 2:**
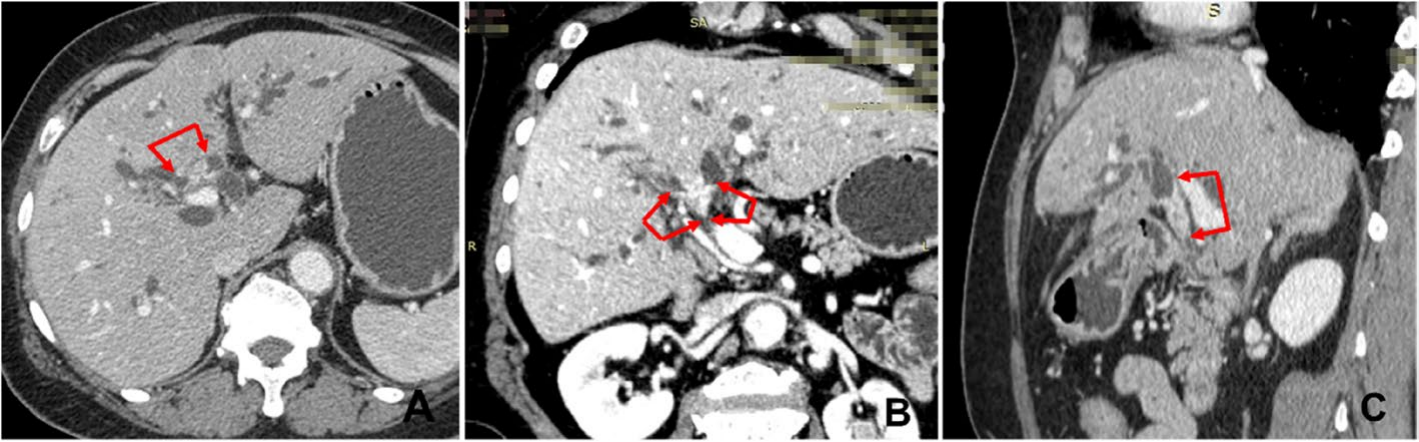
Extent of invasion of hilar cholangiocarcinoma evaluated by multi-slice spiral CT. **A** multi-slice spiral CT image of hilar cholangiocarcinoma. **B** Multi-slice spiral CT image of hilar cholangiocarcinoma MPR phase (coronal section). **C** Multi-slice spiral CT image of hilar cholangiocarcinoma MPR phase (sagittal section)

**Fig. 3:**
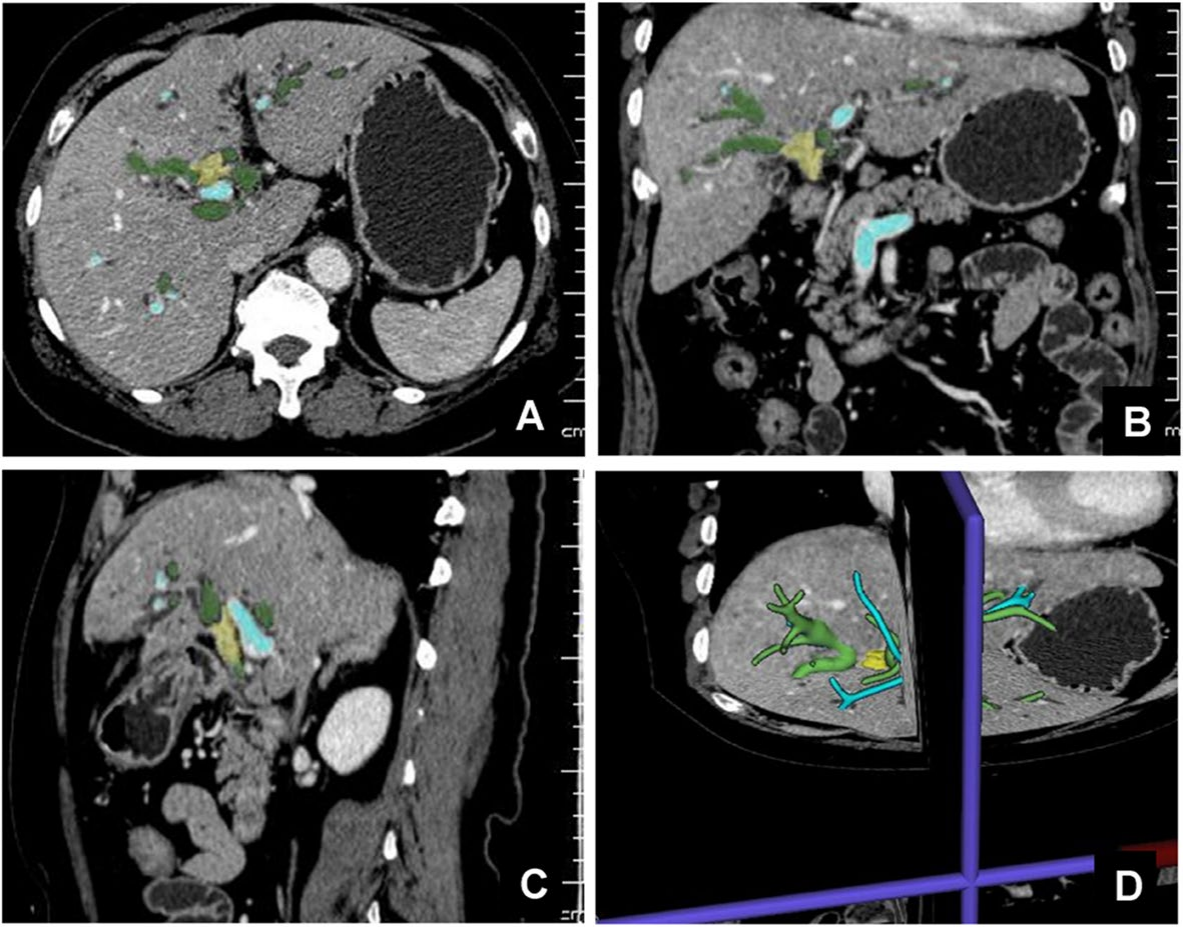
Extent of invasion of hilar cholangiocarcinoma evaluated by 3DVE. **A** 3DVE transverse section of hilar cholangiocarcinoma. **B** 3DVE coronal section of hilar cholangiocarcinoma. **C** 3DVE sagittal section of hilar cholangiocarcinoma. **D** 3DVE restored lesion diagram of hilar cholangiocarcinoma

The previously mentioned surgical resectability factors were respectively evaluated by contrast-enhanced CT (Fig. 2) and 3DVE (Fig. 3), and compared to surgical exploration with pathological examination. According to the Bismuth-Corlette classification, the total accuracy of 3DVE identifying biliary infiltration was 78.4%, compared with 74.8% of CT (Table E3). Furthermore, 3DVE detected the presence of P point involvement, U point involvement, HA invasion, PV invasion, hepatic atrophy, lymph node metastasis, and distant metastasis with accuracies of 92.7%, 93.6%, 84.4%, 81.8%, 96.4%, 80.9%, and 97.3%, respectively, compared with 91.7%, 93.6%, 79.8%, 83.6%, 91.0%, 80.0%, and 97.3% for CT detection, respectively (Tables E4 and E5).

According to the clinicopathological findings and revised criteria for unresectability of hCCA, 86 patients were confirmed resectable (R0/R1 resection), and 25 patients were unresectable. By contrast-enhanced CT, 79 patients were allocated to resectable group and 32 to the unresectable group. According to preoperative 3DVE, 80 patients were resectable and 31 patients belonged to the unresectable group. Compared with CT, 3DVE showed greater sensitivity (90.7%, 95%CI [82.7–95.2%] vs. 83.7%, 95%CI [74.5–90.1%]), specificity (92.0%, 95%CI [75.0–97.8%] vs. 72.0%, 95%CI [52.4–85.7%]), positive predictive value (PPV) (97.5%, 95%CI [91.3–99.3%] vs. 91.1%, 95%CI [82.8–95.6%]), negative predictive value (NPV) (74.2%, 95%CI [56.8–86.3%] vs. 56.3%, 95%CI [39.3–71.8%]), and overall accuracy (91.0%, 95%CI [83.6–95.4%] vs. 81.1%, 95%CI [72.8–87.7%], *p* = 0.033). The agreement value between 3DVE and intraoperative findings with pathological examinations achieved 0.762, higher than that of 0.507 of contrast-enhanced CT (Table 2).

**Table 2:** Comparison of resectability evaluation of hilar cholangiocarcinoma diagnosed by CT and 3DVE

CT evaluation	Intraoperative findings		Sensitivity (95%CI)	Specificity (95%CI)	PPV (95%CI)	NPV (95%CI)	Accuracy (95%CI)	Agreement value (κ)
	Resectable (R0/R1)	Unresectable						
Resectable	72	7	72/86 (83.7, 74.5–90.1)	18/25 (72.0, 52.4–85.7)	72/79 (91.1, 82.8–95.6)	18/32 (56.3, 39.3–71.8)	90/111 (81.1, 72.8–87.7)	0.507
Unresectable	14	18						
Total	86	25						
3DVE evaluation	Intraoperative findings		Sensitivity (95%CI)	Specificity (95%CI)	PPV (95%CI)	NPV (95%CI)	Accuracy (95%CI)	Agreement value (κ)
	Resectable (R0/R1)	Unresectable						
Resectable	78	2	78/86 (90.7, 82.7–95.2)	23/25 (92.0, 75.0–97.8)	78/80 (97.5, 91.3–99.3)	23/31 (74.2, 56.8–86.3)	101/111 (91.0, 83.6–95.4)	0.762
Unresectable	8	23						
Total	86	25						

Unless otherwise indicated, data are number of patients and data in parentheses are percentages

### Hepatic artery involvement was an R1 or R2 resection risk factor in 3DVE

At univariable logistic regression analysis, HA involvement (odds ratio = 3.9, *p* < 0.01) and lymph node metastasis (odds ratio = 2.3, *p* < 0.05) were significantly associated with the absence of R0 resection. The multivariable logistic regression analysis showed that the R0 resection rate of patients without HA involvement in preoperative 3DVE was 81.4% (35 of 43, 95%CI 69.3%, 93.5%), which is significantly higher than 52.9% (36 of 68, 95%CI 40.8%, 65.1%) of the patients with HA involvement. Ultimately, we identified HA involvement as an independent risk factor associated with R1 or R2 resection (adjust odds ratio = 3.5, 95%CI 1.4, 8.8, *p* < 0.01) (Table 3).

**Table 3:** Clinical and 3DVE features associated with R0 resection in patients with hilar cholangiocarcinoma detected at 3D visualization and evaluation

		Resection margin status		Univariable analysis		Multivariable analysis	
Clinical and 3DVE features	Total no. of Patients	R0	R1/R2/ Unresectable	Odds ratio (95% CI)	*P* value	Adjusted odds ratio (95% CI)	*P* value
Age							
> 60 years	83	55(66.3%)	28(33.7%)				
≤ 60 years	28	16(57.1%)	12(42.9%)	1.5(0.6,3.5)	0.386		
Sex							
Male	68	44(64.7%)	24(35.3%)				
Female	43	27(62.8%)	16(37.2%)	1.1(0.5, 2.4)	0.838		
Tumor size							
< 1 cm	4	3(75.0%)	1(25.0%)	1			
1 ~ 3 cm	63	43(68.6%)	20(31.7%)	1.4(0.1,14.3)	0.779		
≥ 3 cm	44	25(56.8%)	19(43.2%)	2.3(0.2, 23.7)	0.490		
Hepatic artery involvement							
Absent	43	35(81.4%)	8(18.6%)				
Present	68	36(52.9%)	32(47.1%)	3.9(1.6, 9.6)	0.003	3.5(1.4, 8.8)	0.008
Portal vein involvement							
Absent	61	41(67.2%)	20(32.8%)				
Present	50	30(60.0%)	20(40.0%)	1.4(0.6.3.0)	0.432		
Bismuth-Corlette Classification							
I	21	13(61.9%)	8(38.1%)				
II	21	15(71.4%)	6(28.6%)	0.7(0.2, 2.4)	0.514		
III	54	33(61.1%)	21(38.9%)	1.0(0.4, 2.9)	0.949		
IV	15	10(66.7%)	5(33.3%)	0.8(0.2, 3.3)	0.769		
Lymph node metastasis							
Absent	50	37(74.0%)	13(26.0%)				
Present	61	34(55.7%)	27(44.3%)	2.3(1.0, 5.1)	0.048	1.9(0.8, 4.3)	0.149
Hepatic atrophy							
Absent	88	58(65.9%)	30(34.1%)				
Present	23	13(56.5%)	10(43.5%)	1.5(0.6, 3.8)	0.405		
P point involvement							
Absent	96	60(62.5%)	36(37.5%)	1	/		
Present	15	11(73.3%)	4(26.7%)	0.6(0.2, 2.0)	0.420		
U point involvement							
Absent	92	62(67.4%)	30(32.6%)				
Present	19	9(47.4%)	10(52.6%)	2.3(0.8, 6.2)	0.103		

Unless otherwise indicated, data are number of patients and data in parentheses are percentages. *CI* confidence interval

### A new 3DVE classification of hilar cholangiocarcinoma

**Fig. 4:**
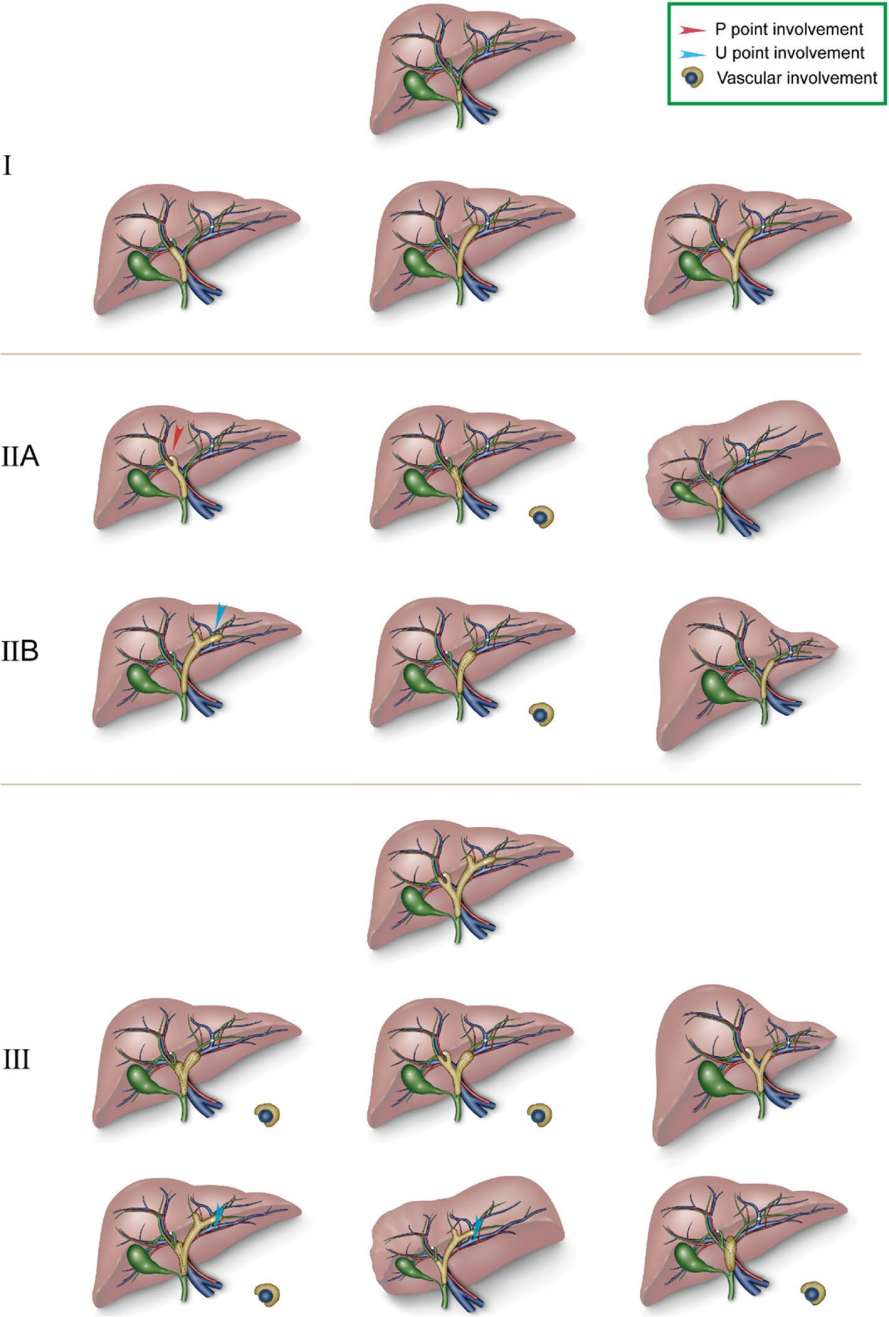
Schematic diagram of preoperative 3DVE classification of hilar cholangiocarcinoma. 3DVE type I: tumor involving common hepatic duct. 3DVE type I: tumor involving hepatic duct confluence and unilateral or bilateral hepatic ducts without involvement of P point and U point, and without vascular involvement or hepatic atrophy. 3DVE type II: tumor involving unilateral P or U point and without, or tumor involving unilateral HA of PV, or unilateral hepatic atrophy, and without contralaterally involving limit of bile duct dissection, HA and PV, and without contralateral hepatic atrophy. 3DVE type III: tumor involving both P and U points; tumor involving unilateral limit of bile duct dissection with contralateral vascular invasion or hepatic atrophy; main portal venous involvement or main hepatic arterial involvement; insufficient FLR volume; metastasis to celiac, portacaval or paraaortic lymph nodes, or distant metastasis

We propose a new 3DVE classification of hCCA based on the key factors influencing resectability for preoperative evaluation (Table E6). In this classification, type I referred to a tumor limited to bile duct without P and U points involvement, vascular invasion, or hepatic atrophy. Type IIA (IIB) was determined for tumor extension to P (U) point, or the right (left) vascular involvement, or the right (left) hepatic atrophy, without U (P) point involvement or left (right) vascular invasion, left (right) hepatic atrophy. Type III included tumor extension to both P point and U point; tumor extension to P point (U point) with left (right) vascular invasion or left (right) hepatic atrophy; or tumor involvement of main PV or proper HA; or metastasis to celiac, portacaval, paraaortic lymph nodes or distant metastasis (Fig. 4, Figure E5). Corresponding therapeutic strategies were recommended for each type of 3DVE classification (Table E7).

The R0 resection rates of hCCA of 3DVE type I, II, and III were 83.9%, 73.5%, and 29.0%, respectively (*p* < 0.001). The R0 resection rates of hCCA of MSKCC T1, T2, and T3 were 73.5%, 69.0%, and 46.9%, respectively (*p* < 0.05) (26). The R0 resection rates based on Bismuth-Corlette classification and AJCC TNM staging system (8th edition) were not statistically significant (Table E8) [32].

**Fig. 5:**
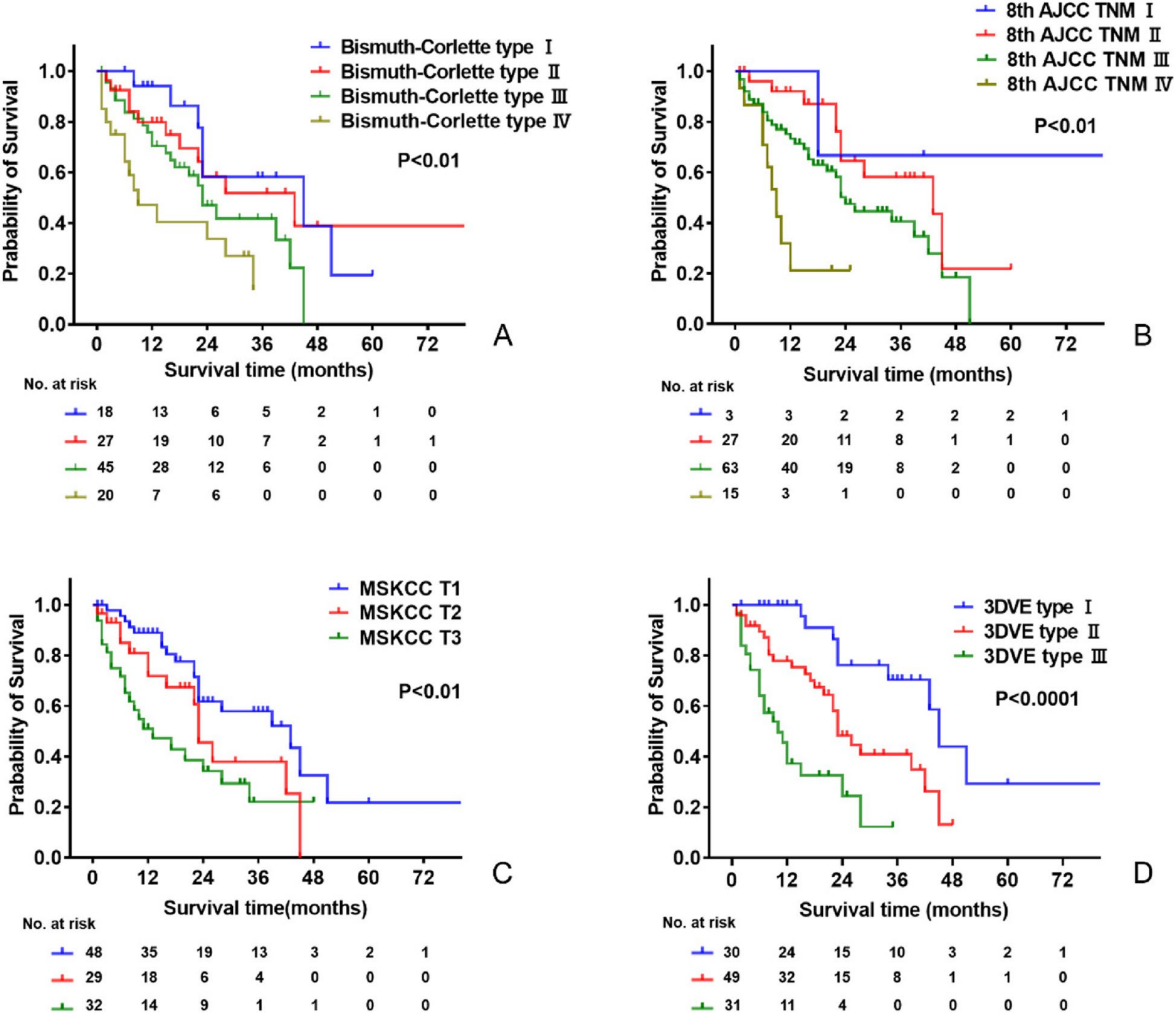
Kaplan–Meier curves for patients with hilar cholangiocarcinoma according to each classification system. **A** Bismuth-Corlette classification system. **B** 8th AJCC TNM system. **C** MSKCC T staging system. **D** 3DVE classification system. Note. 1 patient loosed to follow up. Classifications of 2 patients were not defined in AJCC TNM staging system (8th edition). Classification of 1 patient was not defined in MSKCC T staging system

According to 3DVE classification, the 1-year survival rates were 100%, 77.8%, and 37.2%, respectively, for hCCA patients of type I, II, and III, with corresponding median OS of 45 months, 23 months and 10 months (*p* < 0.0001). For the Bismuth-Corlette system, the 1-year survival rates were 94.1%, 79.8%, 70.5%, and 40.4%, respectively, for type I, II, III, and IV, with corresponding median OS of 45 months,43 months, 23 months, and 9 months (*p* < 0.01). Based on the MSKCC T staging system, the 1-year survival rates were 88.9%, 71.9%, and 51.1%, respectively, for T1, T2, and T3, with corresponding median OS of 43 months, 23 months, and 13 months (*p* < 0.01). In the 8th AJCC TNM staging system, the 1-year survival rate were 66.7%, 92.8%, 73.3%, and 21.3%, respectively, for hCCA patients of TNM I, II, III, and IV, with corresponding median OS of 43 months, 43 months, 24 months, and 9 months (*p* < 0.001) (Fig. 5).

### Analysis of validation cohort

The validation cohort consisted of 34 patients (21 men, 13 women) with a mean age of 63 years ± 11 (range, 33–80 years). The baseline information is shown in Table E9. 3DVE showed a better agreement value than CT in evaluating hCCA resectability (0.718 > 0.343) (Table E10). The R0 resection rate of 3DVE type I, II was 69.2%, 68.8%, significantly higher than 0% of type III (*P* < 0.01). The R0 resection rate was 70.8% in the group without HA involvement, which was significantly higher than 20.0% of HA involvement group (*P* = 0.004) (Table E11).

## Discussion

A preoperative radiological examination is fundamental for the evaluation of surgical resectability and the ultimately selection of patients with a high probability of R0 resection. MRCP, PTC and ERCP could well assess the biliary infiltration [9, 11, 12]. Moreover, through a comprehensive assessment of tumor invasion, hepatic atrophy, and metastasis, CT (or combined with cholangiography) achieved an accuracy of 74.5 ~ 80.5% in evaluating hCCA resectability [10, 13]. In the era of digital medicine, 3D visualization techniques are increasingly embraced as reliable auxiliary tools for liver surgery [21]. In our study, 3DVE exhibited a higher overall accuracy (*P* < 0.05), sensitivity, specificity, NPV, and PPV in evaluating hCCA resectability, compared with those of CT. Specifically, 3DVE exhibited superiority in evaluating biliary infiltration, HA invasion, and hepatic atrophy of hCCA, compared with CT. It also performed similarly to CT in assessing P (U) point involvement and metastasis.

Through 3D reconstruction, stereoscopic and intuitive visual models of biliary tree and blood vessels were rendered. 3DVE enhanced the comprehension of continuity and course of vasculature system, the individualized biliary tree, and the variants and origin of HA [15]. However, the CT sectional images were discontinuous, and required clinicians to transform the 2D images into stereoscopic models by abstract thinking [17, 21]. 3D views could improve our understanding of the spatial relationship between tumor and the branches of biliary tree, which may explain its advantage in evaluating tumor longitudinal infiltration.

Because 3D biliary tree image was extracted in portal venous phase, the P point and U point could be precisely located in reference to the course of portal vein, which facilitates the identification of the important landmarks for surgical planning (Figure E4, Table E6) [17, 28]. Similarly, 3DVE provided accurate assessment of the hepatic atrophy, likely from a full depiction of the liver contour [20]. The tumor contour was reconstructed based on CT portal venous phase, when the HAs were obscurely observed. Through co-registration, HA and the tumor were simultaneously shown in 3D models. 3DVE allows freely rotating the images, and then, HA encasement by tumor could be better viewed and measured [17]. For PV invasion, the diameter of PV is more than HA, which might make the assessment of tumor encasement of PV slightly inaccurate, especially in some cases that tumor contours were irregular and small, where the precise reconstruction of tumor contour was difficult. However, tumor encasement of PV in 2D CT was more likely to be aggressively assessed, as it was based on reviewers’ experience and intuitive thinking. That is the possible reason why evaluation accuracy of PV invasion by 3DVE was lower than that of CT.

R0 margin was a favorable prognostic factor for hCCA [3, 5]. Our research identified that the tumor involvement of HA in preoperative 3DVE is an independent risk factor for R1 or R2 resection of hCCA, which established the association between preoperative 3DVE and surgical resectability. Similar to the previous studies, we validated that HA involvement in hCCA, confirmed by surgery or pathological examinations, was related to advanced stages and a high probability of positive resection margin [33–35].

Several classification systems were widely utilized for predicting prognosis and R0 resection rate of hCCA patients [3–5]. However, the Bismuth-Corlette classification was primarily based on biliary infiltration, and the MSKCC T staging system. In addition, it considered hepatic atrophy and PV involvement, but other hCCA resectability factors were not included [36]. TNM staging has been postoperatively used, depending on the intraoperative and pathological information [37]. Comparatively, the new 3DVE classification considered several parameters, and was distinguished in predicting hCCA resectability and prognosis. Additionally, the 3DVE classification assessed biliary infiltration based on involvement of limits of hepatic ductal resection instead of Bismuth-Corlette classification, which was conducive to the implementation of minor hepatectomy and the preservation of more functional liver volume (Table E6) [30, 31, 38]. In addition, measuring the distance between surgical margin on biliary duct and P (U) points is another guarantee for minor hepatectomy. As minor hepatectomy was not suitable in the case of vascular involvement and hepatic lobar atrophy, 3DVE that accurately evaluated varied resectability parameters could be a useful radiological tool to improve clinical decision-making.

Besides preoperative diagnosis, Fang et al. [39] and Zhang et al. [40] demonstrated the 3D-aided surgery for hepatocellular carcinoma (HCC), compared with non-3D radiological evaluation, is linked to shorter operation time, lower hepatic inflow occlusion rate, less bleeding volume, and reduced postoperative complications. What is more, 3D fusion image navigation system based on ultrasound has elevated efficacy of percutaneous microwave ablation the complete ablation rate for HCC treatment [41]. Therefore, further study is needed to explore the potential value of 3D visualization and the derived tools for hCCA treatment.

Our study had some limitations. First, it is a single-center, retrospective study with inherent biases. Multicentral data could be included and compared, since a prospective study may further evaluate the efficacy of the 3DVE system. Second, the 3D reconstruction was the secondary processing of images originating from raw images, and thus, the 3D reconstruction could not provide additional information. The quality of 3D visualization models was also influenced by the experience of the manipulators. Third, as slight bile duct dilation was not clearly differentiated in CT, 3D reconstruction might fail to apply to hCCA without an obvious bile duct dilatation. Fourth, the multiple variables analysis was based on the 3DVE, and not all the odds rates of variables were statistically significant, so we did not formulate a classification entirely based on the odds rates or even determined each stage. Thus, our classification was formulated based on combination of the canonical hCCA classification and clinical practice.

In conclusion, 3DVE exhibited a significantly higher accuracy in the preoperative evaluation of hCCA resectability in comparison to contrast-enhanced CT. The tumor involvement of HA in 3DVE was identified as the independent risk factor for the absence of R0 margin of hCCA. Additionally, the proposed preoperative 3DVE classification system performed well in predicting R0 resection rate and prognosis of hCCA patients.

### Supplementary Information

**Additional file 1:** **Table E1.** Result of clinical classification of 111 patients of hilar cholangiocarcinoma. **Table E2.** Resection radicality according to surgical procedures of 111 patients of hilar cholangiocarcinoma. **Table E3.** Evaluation of tumor longitudinal infiltration by CT/3DVE and intraoperative findings combined with pathological examination. **Table E4.** CT evaluation of resectability factors in hilar cholangiocarcinoma. **Table E5.** 3D visualization and evaluation of resectability factors in hilar cholangiocarcinoma. **Table E6.** Contents of classification systems of hilar cholangiocarcinoma. **Table E7.** Clinical 3DVE classification of hilar cholangiocarcinoma. **Table E8.** Percentage of R0 resection according to different staging systems of 111 patients of hilar cholangiocarcinoma. **Table E9.** Clinicopathological features of validation cohort. **Table E10.** Resectability evaluated by CT and 3DVE in validation cohort. **Table E11.** R0 resection rate in validation cohort.

**Additional file 2:** **Figure E1.** Restoration of 3D structure of lesions of hilar cholangiocarcinoma and adjustment of tumor border in 2D images. **Figure E2.** Simulation of resection of right anterior lobe of the liver and resection effect. **Figure E3.** 3D visualization model of hilar cholangiocarcinoma according to Bismuth-Corlette classification. **Figure E4.** 3D visualization models to measure the distance between P and U points and tumor border. **Figure E5.** Actual reconstruction diagrams of 3DVE classification of hilar cholangiocarcinoma.

## Data Availability

The datasets analyzed during the current study are available from the corresponding author on reasonable request.

## Abbreviations

CT: Computed tomography
ERC: Endoscopic retrograde cholangiography
hCCA: Hilar cholangiocarcinoma
HA: Hepatic artery
MRCP: Magnetic resonance cholangiopancreatography
MRI: Magnetic resonance imaging
PTC: Percutaneous transhepatic cholangiography
PV: Portal vein
3DVE: Three-dimensional visualization and evaluation
