# Supplementary material for: Three-dimensional visualization and evaluation of hilar cholangiocarcinoma resectability and proposal of a new classification
Source: World J Surg Oncol. 2023 Aug 5;21:239. doi: 10.1186/s12957-023-03126-2 (PMC10403901; doi:10.1186/s12957-023-03126-2)
Supplement: Supplementary file 1 — Additional file 1: Table E1. Result of clinical classification of 111 patients of hilar cholangiocarcinoma. Table E2. Resection radicality according to surgical procedures of 111 patients of hilar cholangiocarcinoma. Table E3. Evaluation of tumor longitudinal infiltration by CT/3DVE and intraoperative findings combined with pathological examination. Table E4. CT evaluation of resectability factors in hilar cholangiocarcinoma. Table E5. 3D visualization and evaluation of resectability factors in hilar cholangiocarcinoma. Table E6. Contents of classification systems of hilar cholangiocarcinoma. Table E7. Clinical 3DVE classification of hilar cholangiocarcinoma. Table E8. Percentage of R0 resection according to different staging systems of 111 patients of hilar cholangiocarcinoma. Table E9. Clinicopathological features of validation cohort. Table E10. Resectability evaluated by CT and 3DVE in validation cohort. Table E11. R0 resection rate in validation cohort. [file 12957_2023_3126_MOESM1_ESM.docx]

**Supplementary Tables**

**Table E1:** **Result of clinical classification of 111 patients of hilar cholangiocarcinoma**

| Clinical classification | Resectability by three-dimensional visualization evaluation | | | |
| --- | --- | --- | --- | --- |
|  | Total | Resectable group | Unresectable group | P value |
| No. of patients | 111 | 80 | 31 |  |
| Bismuth-Corlette classification |  |  |  | <0.05 |
| Ⅰ | 19(17.1%) | 18(22.5%) | 1(3.2%) |  |
| Ⅱ | 27(24.3) | 21(26.3%) | 6(19.4%) |  |
| Ⅲ | 45(40.5%) | 29(36.3%) | 16(51.6%) |  |
| Ⅳ | 20(18.0%) | 12(15.0%) | 8(25.8%) |  |
| MSKCC T staging |  |  |  | <0.001 |
| T1 | 49(44.1%) | 45(56.3%) | 4(12.9%) |  |
| T2 | 29(26.1%) | 21(26.3%) | 8(25.8%) |  |
| T3 | 32(28.8%) | 14(17.5%) | 18(58.1%) |  |
| Not defined | 1(0.9%) | 0(0.0%) | 1(3.2%) |  |
| AJCC tumor category (8^th^ edition) |  |  |  | <0.001 |
| Tx | 2(1.8%) | 0(0.0%) | 2(6.5%) |  |
| T1 | 4(3.6%) | 3(3.8%) | 1(3.2%) |  |
| T2 | 38(34.2%) | 37(46.3%) | 1(3.2%) |  |
| T3 | 35(31.5%) | 29(36.3%) | 6(19.4%) |  |
| T4 | 32(28.8%) | 11(13.8%) | 21(67.7%) |  |
| AJCC node category (8^th^ edition) |  |  |  | <0.001 |
| Nx | 4(3.6%) | 1(1.3%) | 3(9.7%) |  |
| N0 | 53(47.7%) | 48(60.0%) | 5(16.1%) |  |
| N1 | 42(37.8%) | 28(35.0) | 14(45.2%) |  |
| N2 | 12(10.8%) | 3(3.8%) | 9(29.0%) |  |
| AJCC metastasis category  (8^th^ edition) |  |  |  | <0.01 |
| M0 | 107(96.4%) | 80(100.0%) | 27(87.1%) |  |
| M1 | 4(3.6%) | 0(0.0%) | 4(12.9%) |  |
| AJCC tumor stage (8^th^ edition) |  |  |  | <0.001 |
| Ⅰ | 3(2.7%) | 3(3.8%) | 0(0.0%) |  |
| Ⅱ | 27(24.3%) | 26(32.5%) | 1(3.2%) |  |
| Ⅲ | 64(57.7%) | 47(58.8%) | 17(54.8%) |  |
| Ⅳ | 15(13.5%) | 4(5.0%) | 11(35.5%) |  |
| Not defined | 2(1.8%) | 0(0.0%) | 2(6.5%) |  |

Note. — Data are presented as number (%) or mean ± standard deviation.

**Table E2:** **Resection radicality according to surgical procedures of 111 patients of hilar cholangiocarcinoma**

| Bismuth-Corlette Classification | Ⅰ | | | |  | Ⅱ | |  | Ⅲa | |  | Ⅲb | |  | Ⅳ | |
| --- | --- | --- | --- | --- | --- | --- | --- | --- | --- | --- | --- | --- | --- | --- | --- | --- |
| Surgical procedures | All | | | R0 |  | All | R0 |  | All | R0 |  | All | R0 |  | All | R0 |
| Minor hepatectomy | 18 | | | 13  (72.2) |  | 15 | 12  (80.0) |  | 4 | 1  (25.0) |  |  |  |  | 1 | 1  (100.0) |
| Left hemihepatectomy |  | | |  |  | 4 | 4  (100.0) |  | 2 | 1  (50.0) |  |  |  |  | 8 | 8  (100.0) |
| Right hemihepatectomy | |  | |  |  | 2 | 1  (50.0) |  | 18 | 15  (83.3) |  | 8 | 7  (87.5) |  | 4 | 3  (75.0) |
| Right trisegmentectomy | |  | |  |  |  |  |  |  |  |  |  |  |  | 1 | 1  (100.0) |
| Special hepatectomy | 1 | | | 1  (100.0) |  | 2 | 2  (100.0) |  | 1 | 1  (100.0) |  |  |  |  |  |  |
| Liver transplantation |  | | |  |  |  |  |  | 2 | / |  |  |  |  | 1 | / |
| Internal/external biliary drainage |  | | |  |  | 2 | / |  | 3 | / |  | 1 | / |  | 2 | / |
| Exploratory laparotomy |  | | |  |  | 2 | / |  | 2 | / |  |  |  |  | 2 | / |
| Palliative resection |  | | |  |  |  |  |  | 1 | / |  | 3 | / |  | 1 | / |
| Total | 19 | | | 14 |  | 27 | 19 |  | 33 | 18 |  | 12 | 7 |  | 20 | 13 |
| Radical resection rate（%） | | | 73.7 | |  | 70.4 | |  | 54.5 | |  | 58.3 | |  | 65.0 | |

Note. — Unless otherwise indicated, data are number of patients and data in parentheses are percentages.

**Table E3:** **Evaluation of tumor longitudinal infiltration by CT/3DVE and intraoperative findings combined with pathological examination**

| CT evaluation | Intraoperative findings combined with pathological examination (Bismuth-Corlette types) | | | | | Total | Interobserver agreement (κvalue) |
| --- | --- | --- | --- | --- | --- | --- | --- |
|  | Ⅰ | Ⅱ | Ⅲa | Ⅲb | Ⅳ |  |  |
| Ⅰ | **17** | 1 | 1 | 0 | 0 | 19 |  |
| Ⅱ | 2 | **18** | 3 | 0 | 2 | 25 |  |
| Ⅲa | 0 | 5 | **26** | 1 | 2 | 34 |  |
| Ⅲb | 0 | 3 | 1 | **10** | 4 | 18 |  |
| Ⅳ | 0 | 0 | 2 | 1 | **12** | 15 |  |
| Total | 19 | 27 | 33 | 12 | 20 | 111 |  |
| Accuracy (%) | 89.5 | 66.7 | 78.8 | 83.3 | 60.0 | 74.8 | 0.517 |
| 3DVE evaluation | Intraoperative findings combined with pathological examination (Bismuth-Corlette type) | | | | | Total | Interobserver agreement (κvalue) |
|  | Ⅰ | Ⅱ | Ⅲa | Ⅲb | Ⅳ |  |  |
| Ⅰ | **17** | 3 | 1 | 0 | 0 | 21 |  |
| Ⅱ | 2 | **16** | 1 | 0 | 2 | 21 |  |
| Ⅲa | 0 | 4 | **29** | 0 | 2 | 35 |  |
| Ⅲb | 0 | 3 | 1 | **12** | 3 | 19 |  |
| Ⅳ | 0 | 1 | 1 | 0 | **13** | 15 |  |
| Total | 19 | 27 | 33 | 12 | 20 | 111 |  |
| Accuracy (%) | 89.5 | 59.3 | 87.9 | 100.0 | 65.0 | 78.4 | 0.646 |

Note. — Unless otherwise indicated, data are number of patients.

**Table E4:** **CT evaluation of resectability factors in hilar cholangiocarcinoma**

| CT | Intraoperative diagnosis | | | | | | | | Sensitivity (%) | Specificity (%) | NPV (%) | PPV (%) | Accuracy (95%CI) | Interobserver agreement (κvalue) |
| --- | --- | --- | --- | --- | --- | --- | --- | --- | --- | --- | --- | --- | --- | --- |
|  | present | | | absent | | | | |  |  |  |  |  |  |
| P point involvement^δ^ | | |  | |  | | | | 13/19  (68.4) | 87/90  (96.7) | 13/16  (81.3) | 87/93  (93.5) | 100/109  (91.7, 84.5-95.9) | 0.594 |
| Present | | 13 | | | 3 | | | |  |  |  |  |  |  |
| Absent | | 6 | | | 87 | | | |  |  |  |  |  |  |
| U point involvement^θ^ | | |  | |  | | | | 17/19  (89.5) | 85/90  (94.4) | 17/22  (77.3) | 85/87  (97.7) | 102/109  (93.6, 86.8-97.1) | 0.637 |
| Present | | 17 | | | 5 | | | |  |  |  |  |  |  |
| Absent | | 2 | | | 85 | | | |  |  |  |  |  |  |
| Hepatic artery invasion^ε^ | | | | | | |  | | 48/57  (84.2) | 39/52  (75.0) | 48/61  (78.7) | 39/48  (81.3) | 87/109  (79.8, 70.8-86.7) | 0.721 |
| Present | | 48 | | | 13 | | | |  |  |  |  |  |  |
| Absent | | 9 | | | 39 | | | |  |  |  |  |  |  |
| Portal vein invasion^ζ^ | | | | | |  | |  | 44/55  (80.0) | 48/55  (87.2) | 44/51  (86.3) | 48/59  (81.4) | 92/110  (83.6, 75.1-89.8) | 0.655 |
| Present | | 44 | | | 7 | | | |  |  |  |  |  |  |
| Absent | | 11 | | | 48 | | | |  |  |  |  |  |  |
| Hepatic lobar atrophy | | | | | |  | |  | 14/21  (66.7) | 87/90  (96.7) | 14/17  (82.4) | 87/94  (92.6) | 101/111  (91.0, 83.7-95.4) | 0.517 |
| Present | | 14 | | | 3 | | | |  |  |  |  |  |  |
| Absent | | 7 | | | 87 | | | |  |  |  |  |  |  |
| Lymph node metastasis^η^ | | | | | |  | |  | 47/54  (87.0) | 41/56  (73.2) | 47/62  (75.8) | 41/48  (85.4) | 88/110  (80.0, 71.1-86.8) | 0.722 |
| Present | | 47 | | | 15 | | | |  |  |  |  |  |  |
| Absent | | 7 | | | 41 | | | |  |  |  |  |  |  |
| Distant metastasis | | | | | |  | |  | 3/5  (60.0) | 105/106  (99.1) | 3/4  (75.0) | 105/107  (98.1) | 108/111  (97.3, 91.7-99.3) | 0.635 |
| Present | | 3 | | | 1 | | | |  |  |  |  |  |  |
| Absent | | 2 | | | 105 | | | |  |  |  |  |  |  |

Note. — Unless otherwise indicated, data are number of patients and data in parentheses are percentages.

δ 2 cases were not detected the presence of P point involvement in CT without pathological confirmation.

θ 1 case was detected the presence of U point involvement and the other one was detected the absence of U point involvement in CT without pathological confirmation.

ε 2 cases were detected the presence of hepatic artery involvement in CT without pathological confirmation.

ζ 1 case was detected the presence of portal vein involvement in CT without pathological confirmation.

η 1 case was detected the absence of lymph node metastasis in CT without pathological confirmation.

**Table E5: 3D visualization and evaluation of resectability factors in hilar cholangiocarcinoma**

| 3DVE | Intraoperative diagnosis | | | | | | | | Sensitivity (%) | Specificity (%) | NPV (%) | PPV (%) | Accuracy (95%CI) | Interobserver agreement (κvalue) |
| --- | --- | --- | --- | --- | --- | --- | --- | --- | --- | --- | --- | --- | --- | --- |
|  | present | | | absent | | | | |  |  |  |  |  |  |
| P point involvement^δ^ | | |  | |  | | | | 13/19  (68.4) | 88/90  (97.8) | 13/15  (86.7) | 88/94  (93.6) | 101/109  (92.7, 85.6-96.6) | 0.830 |
| Present | | 13 | | | 2 | | | |  |  |  |  |  |  |
| Absent | | 6 | | | 88 | | | |  |  |  |  |  |  |
| U point involvement^θ^ | | |  | |  | | | | 15/19  (78.9) | 87/90  (96.7) | 15/18  (83.3) | 87/91  (95.6) | 102/109  (93.6, 86.8-97.1) | 0.755 |
| Present | | 15 | | | 3 | | | |  |  |  |  |  |  |
| Absent | | 4 | | | 87 | | | |  |  |  |  |  |  |
| Hepatic artery invasion^ε^ | | | | | | |  | | 53/57  (93.0) | 39/52  (75.0) | 53/66  (80.3) | 39/43  (90.7) | 92/109  (84.4, 75.9-90.4) | 0.774 |
| Present | | 53 | | | 13 | | | |  |  |  |  |  |  |
| Absent | | 4 | | | 39 | | | |  |  |  |  |  |  |
| Portal vein invasion^ζ^ | | | | | |  | |  | 42/55  (76.4) | 48/55  (87.3) | 42/49  (85.7) | 48/61  (78.7) | 90/110  (81.8, 73.1-88.3) | 0.726 |
| Present | | 42 | | | 7 | | | |  |  |  |  |  |  |
| Absent | | 13 | | | 48 | | | |  |  |  |  |  |  |
| Hepatic lobar atrophy | | | | | |  | |  | 20/21  (95.2) | 87/90  (96.7) | 20/23  (87.0) | 87/88  (98.9) | 107/111  (96.4, 90.5-98.8) | 0.661 |
| Present | | 20 | | | 3 | | | |  |  |  |  |  |  |
| Absent | | 1 | | | 87 | | | |  |  |  |  |  |  |
| Lymph node metastasis^η^ | | | | | |  | |  | 47/54  (87.0) | 42/56  (75.0) | 47/61  (77.0) | 42/49  (85.7) | 89/110  (80.9, 72.1-87.5) | 0.667 |
| Present | | 47 | | | 14 | | | |  |  |  |  |  |  |
| Absent | | 7 | | | 42 | | | |  |  |  |  |  |  |
| Distant metastasis | | | | | |  | |  | 3/5  (60.0) | 105/106  (99.1) | 3/4  (75.0) | 105/107  (98.1) | 108/111  (97.3, 91.7-99.3) | 0.635 |
| Present | | 3 | | | 1 | | | |  |  |  |  |  |  |
| Absent | | 2 | | | 105 | | | |  |  |  |  |  |  |

Note. — Unless otherwise indicated, data are number of patients and data in parentheses are percentages.

δ 2 cases were detected the absence of P point involvement in 3DVE without pathological confirmation.

θ 1 case was detected the presence of U point involvement and the other one was detected the absence of U point involvement in 3DVE without pathological confirmation.

ε 2 cases were detected the presence of hepatic artery involvement in 3DVE without pathological confirmation.

ζ 1 case was detected the presence of portal vein involvement in 3DVE without pathological confirmation.

η 1 case was detected the absence of lymph node metastasis in 3DVE without pathological confirmation.

**Table E6:** **Contents of classification systems of hilar cholangiocarcinoma**

| Classification | Evaluating Comments | | | | | | | |
| --- | --- | --- | --- | --- | --- | --- | --- | --- |
|  | Biliary infiltration | P/U point involvement | HA invasion | PV invasion | Hepatic atrophy | LN metastasis | Distant metastasis | FLR volume |
| Bismuth-Corlette | **√** | **/** | **/** | **/** | **/** | **/** | **/** | **/** |
| MSKCC T | **√** | **/** | **/** | **√** | **√** | **/** | **/** | **/** |
| AJCC TNM staging system (8^th^ edition) | **√** | **/** | **√** | **√** | **/** | **√** | **√** | **/** |
| 3DVE | **√** | **√** | **√** | **√** | **√** | **√** | **√** | **√** |

Note- LN means lymph node.

**Table E7: Clinical 3DVE classification of hilar cholangiocarcinoma**

| Classification | | Revised Criteria | Recommend surgical procedures |
| --- | --- | --- | --- |
| Type Ⅰ | Tumor infiltrating common hepatic duct or tumor involving unilateral or bilateral hepatic ducts but not involving P point and U point, and without involvement of hepatic artery or portal vein, and without hepatic lobe atrophy | | Minor hepatectomy + S1 |
| Type Ⅱ | Tumor mainly involving unilateral limit of bile duct dissection, or unilateral hepatic arterial or portal vein invasion, or hepatic atrophy | |  |
| Type ⅡA | Tumor involvement of P point, or right vascular invasion or right hepatic lobar atrophy, and without U point involvement, left vascular invasion, or left hepatic atrophy | | Right hepatectomy/ trisegmentectomy + S1 |
| Type ⅡB | Tumor involvement of U point, or left vascular invasion or left hepatic lobar atrophy, and without P point involvement, right vascular invasion, or right hepatic atrophy | | Left hepatectomy/ trisegmentectomy + S1 |
| Type Ⅲ | Tumor extension to both P point and U point or bilateral vascular invasion, or hepatic atrophy;  Tumor extension to P point and left vascular invasion or left hepatic lobe atrophy;  Tumor extension to U point and right vascular invasion or right hepatic lobe atrophy;  Main portal venous involvement or main hepatic arterial involvement;  Insufficient FLR volume. | | Neoadjuvant radiotherapy or chemotherapy |

**Table E8:** **Percentage of R0 resection according to different staging systems of 111 patients of hilar cholangiocarcinoma**

| Clinical stage | Intraoperative findings combined with pathological examination | | | P value |
| --- | --- | --- | --- | --- |
|  | R0 | R1/R2/ Unresectable | R0 resection rate（%） |  |
| No. of patients | 71 | 40 | 64.0 |  |
| Bismuth-Corlette classification |  |  |  | 0.288 |
| Ⅰ | 14 (19.7%) | 5 (12.5%) | 73.7 |  |
| Ⅱ | 19 (26.8%) | 8 (20.0%) | 70.4 |  |
| Ⅲ | 25 (35.2%) | 20 (50.0%) | 55.6 |  |
| Ⅳ | 13 (18.3%) | 7 (17.5%) | 65.0 |  |
| MSKCC T staging |  |  |  | <0.05 |
| T1 | 36 (50.7%) | 13 (32.5%) | 73.5 |  |
| T2 | 20 (28.2%) | 9 (22.5%) | 69.0 |  |
| T3 | 15 (21.1%) | 17 (42.5%) | 46.9 |  |
| Not defined | 0 (0.0%) | 1 (2.5%) | / |  |
| AJCC TNM staging (8^th^ edition) |  |  |  | 0.209 |
| Ⅰ | 3 (4.2%) | 0 (0.0%) | 100.0 |  |
| Ⅱ | 20 (28.2%) | 7 (17.5%) | 74.1 |  |
| Ⅲ | 38 (53.5%) | 26 (65%) | 59.4 |  |
| Ⅳ | 10 (14.1%) | 5 (12. 5%) | 66.7 |  |
| Not defined | 0 (0.0%) | 2 (5.0%) | / |  |
| 3DVE classification |  |  |  | <0.001 |
| Ⅰ | 26 (36.6%) | 5 (12.5%) | 83.9 |  |
| Ⅱ | 36 (50.7%) | 13 (32.5%) | 73.5 |  |
| Ⅲ | 9 (12.7%) | 22 (55.0%) | 29.0 |  |

Note. — Data are presented as number (%) or mean ± standard deviation.

**Table E9:** **Clinicopathological features of validation cohort**

|  | Resectability by three-dimensional visualization evaluation | | | |
| --- | --- | --- | --- | --- |
| Clinicopathological features | Total | Resectable group | Unresectable group | P value |
| No. of patients | 34 | 29 | 5 |  |
| Age (y)* | 63±11 | 64±10 | 57±13 | 0.434 |
| Sex (M/F) | 21/13 | 18/11 | 3/2 | 0.930 |
| Preoperative biliary drainage | 23(67.6%) | 8(27.9%) | 3(60.0%) | 0.152 |
| Tumor size（≤1/1~3cm/≥3cm） | 5/19/9 | 5/17/7 | 0/2/2 | 0.450 |
| Tumor form |  |  |  | 0.506 |
| Sclerosing | 10 | 8 | 2 |  |
| Mass | 7 | 7 | 0 |  |
| Polypoid | 4 | 4 | 0 |  |
| Mixed | 12 | 10 | 2 |  |
| Tumor differentiation (well/moderately/poorly) | 6/25/2 | 6/22/1 | 0/3/1 | 0.172 |
| Hepatic artery involvement | 11 (33.3%) | 7 (24.1%) | 4 (100.0%) | 0.003 |
| Portal vein involvement | 12 (36.4%) | 8 (27.6%) | 4 (100.0%) | 0.005 |
| Hepatic vein involvement | 2 (6.06%) | 1 (3.40%) | 1 (25.0%) | 0.090 |
| Lymph node metastasis | 15 (45.5%) | 13 (44.8%) | 2 (50.0%) | 0.846 |
| Distant metastasis | 3 (8.8%) | 2 (6.9%) | 1 (25.0%) | 0.340 |
| Perineural invasion | 21 (63.6%) | 18 (62.1%) | 3 (75.0%) | 0.614 |
| Hepatic lobe atrophy | 13 (39.4%) | 10 (34.5%) | 3 (75.0%) | 0.120 |
| Resection status |  |  |  | <0.001 |
| R0 | 20(58.8%) | 20(74.1%) | 0(0.0%) |  |
| R1 | 6(17.6%) | 6(22.2%) | 0(0.0%) |  |
| R2 | 0(0.0%) | 0(0.0%) | 0(0.0%) |  |
| Unresectable | 8(23.5%) | 3(3.7%) | 5(100.0%) |  |

Note. — Data are presented as number (%) or mean ± standard deviation.

A patient belonged to unresectable group due to distant metastasis, and the clinicopathological information of HA involvement, PV involvement, hepatic vein involvement, lymph node metastasis, perineural invasion and hepatic lobe atrophy was not documented. Three patients were detected the absence of information of tumor differentiation.

**Table E10:** **Resectability evaluated by CT and 3DVE in validation cohort**

| CT evaluation | Intraoperative findings | | Sensitivity  (95%CI) | Specificity  (95%CI) | PPV  (95%CI) | NPV  (95%CI) | Accuracy (95%CI) | Agreement value (κ) |
| --- | --- | --- | --- | --- | --- | --- | --- | --- |
|  | Resectable（R0/R1） | Unresectable |  |  |  |  |  |  |
| Resectable | 24 | 5 | 24/26  (85.7, 68.5-94.3) | 3/8  (37.5, 13.7-69.4) | 24/29  (82.8, 65.6-92.4) | 3/5  (60.0, 23.1-88.2) | 27/34  (79.4, 63.2-89.7) | 0.343 |
| Unresectable | 2 | 3 |  |  |  |  |  |  |
| Total | 26 | 8 |  |  |  |  |  |  |
| 3DVE evaluation | Intraoperative findings | | Sensitivity (95%CI) | Specificity (95%CI) | PPV (95%CI) | NPV (95%CI) | Accuracy (95%CI) | Agreement value (κ) |
|  | Resectable（R0/R1） | Unresectable |  |  |  |  |  |  |
| Resectable | 26 | 3 | 26/26  (100, 87.1-100) | 5/8  (62.5, 30.6-86.3) | 26/29  (89.7, 73.6-96.4) | 5/5  (100, 56.6-100) | 31/34  (91.2, 77.0-97.0) | 0.718 |
| Unresectable | 0 | 5 |  |  |  |  |  |  |
| Total | 26 | 8 |  |  |  |  |  |  |

**Table E11:** **R0 resection rate in validation cohort**

|  | Intraoperative findings combined with pathological examination | | | | P value |
| --- | --- | --- | --- | --- | --- |
|  | R0 | | R1/R2/ Unresectable | R0 resection rate（%） |  |
| HA involvement |  | |  |  | 0.004 |
| Present | 2(10.5) | | 8(57.1) | 20.0 |  |
| Absent | 17(89.5) | | 6(42.9) | 73.9 |  |
| Preoperative 3DVE classification |  |  | |  |  |
| Ⅰ | 9(45.0) | 4(28.6) | | 69.2 | 0.009^θ^ |
| Ⅱ | 11(55.0) | 5(35.7) | | 68.8 | 0.007^δ^ |
| Ⅲ | 0(0.0) | 5(35.7) | | 0.0 |  |

θ Statistical difference was calculated in 3DVE classification Ⅰ and Ⅲ.

δ Statistical difference was calculated in 3DVE classification Ⅱ and Ⅲ.
