# Supplementary material for: Three-dimensional visualization and evaluation of hilar cholangiocarcinoma resectability and proposal of a new classification
Source: World J Surg Oncol. 2023 Aug 5;21:239. doi: 10.1186/s12957-023-03126-2 (PMC10403901; doi:10.1186/s12957-023-03126-2)
Supplement: Supplementary file 2 — Additional file 2: Figure E1. Restoration of 3D structure of lesions of hilar cholangiocarcinoma and adjustment of tumor border in 2D images. Figure E2. Simulation of resection of right anterior lobe of the liver and resection effect. Figure E3. 3D visualization model of hilar cholangiocarcinoma according to Bismuth-Corlette classification. Figure E4. 3D visualization models to measure the distance between P and U points and tumor border. Figure E5. Actual reconstruction diagrams of 3DVE classification of hilar cholangiocarcinoma. [file 12957_2023_3126_MOESM2_ESM.docx]

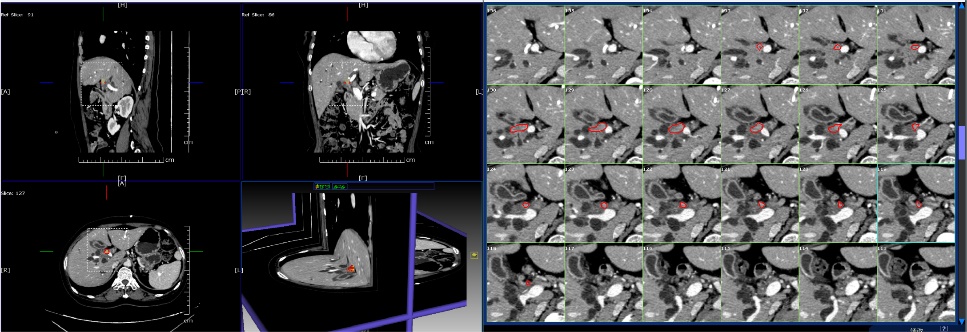


**Figure E1:** Restoration of 3D structure of lesions of hilar cholangiocarcinoma and adjustment of tumor border in 2D images.


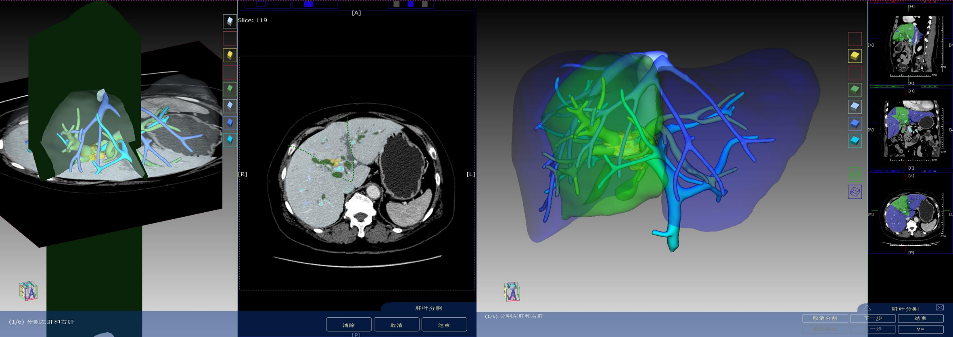


**Figure E2:** Simulation of resection of right anterior lobe of the liver and resection effect.


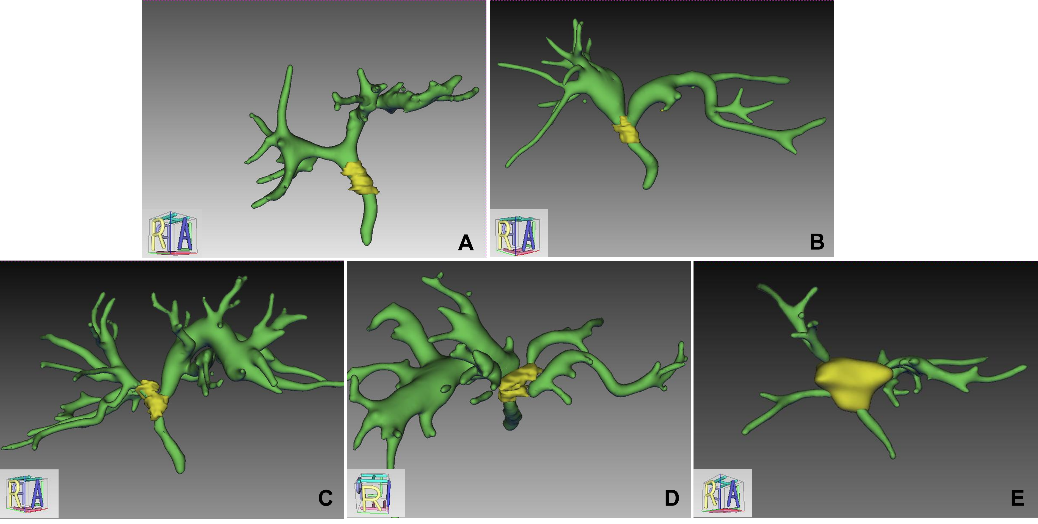


**Figure E3:** 3D visualization model of hilar cholangiocarcinoma according to Bismuth-Corlette classification.

1. Type Ⅰ. B. Type Ⅱ. C. Type Ⅲa. D. Type Ⅲb. E. Type Ⅳ.


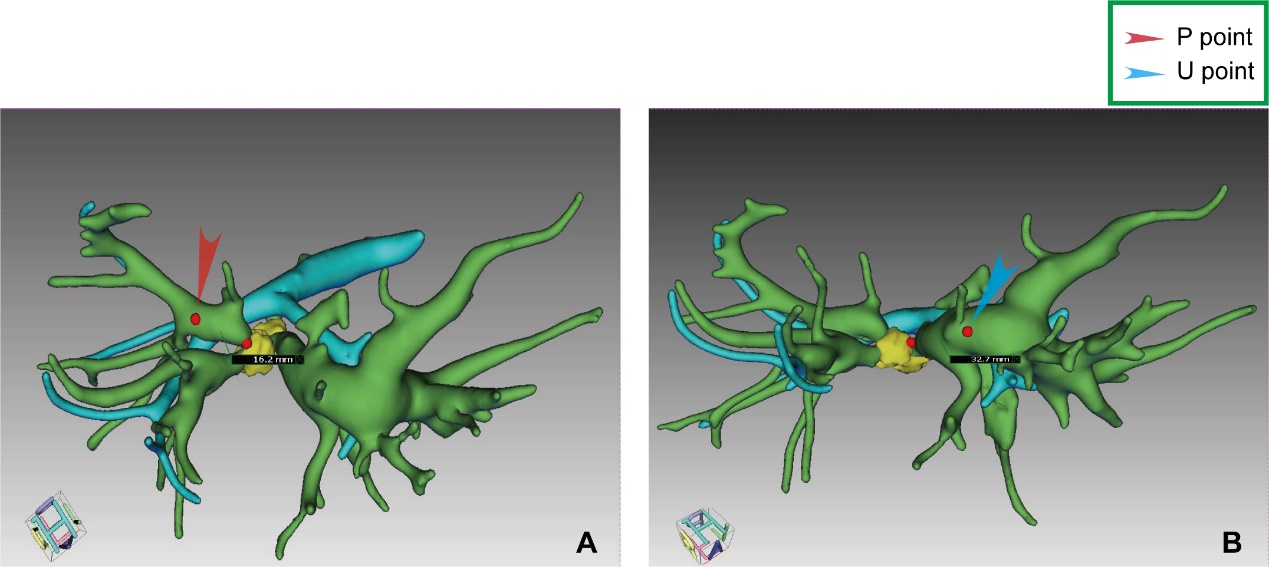


**Figure E4:** 3D visiualization models to measure the distance between P and U points and tumor border.

1. Distance between P point and right border of tumor. B. Distance between U point and left border of tumor.


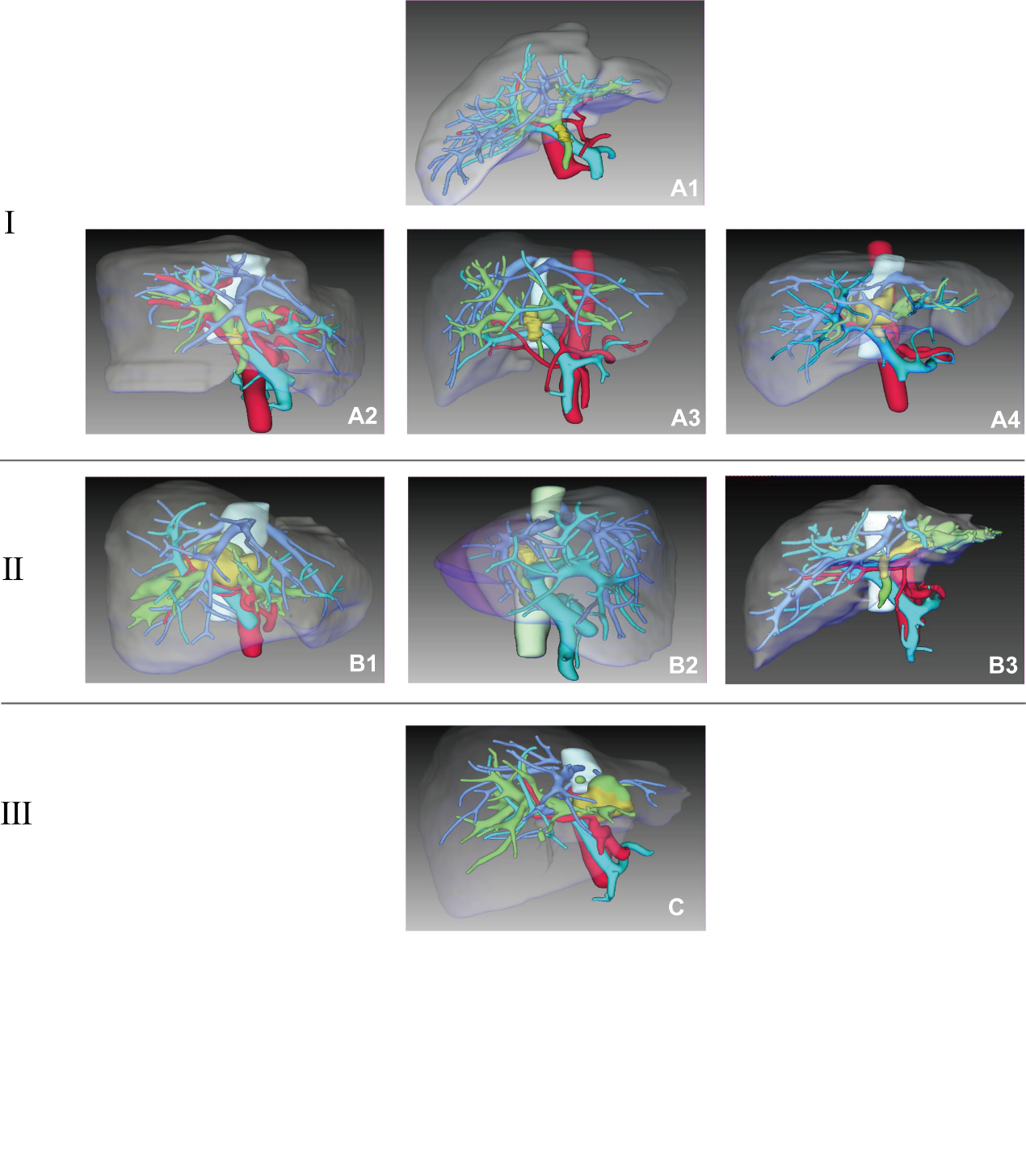
 **Figure E5:** Actual reconstruction diagrams of 3DVE classification of hilar cholangiocarcinoma.

3DVE type Ⅰ: tumor involving common hepatic duct (A1); tumor involving hepatic duct confluence (A2); tumor involving unilateral (A3) or bilateral (A4) hepatic duct without P (U) point involvement, and without vascular invasion or hepatic atrophy or invasion. 3DVE Type Ⅱ: tumor involving unilateral limit of hepatic ductal transection (B1, P point involvement) or unilateral vascular involvement (B2, B3) or hepatic atrophy (B2). 3DVE Type Ⅲ: tumor involving unilateral limit of ductal resection (U point) and proper hepatic artery.
